# Regression, kinetics and isotherm models for biosorption of organic pollutants, suspended and dissolved solids by environmentally friendly and economical dried *Phragmites australis*†

**DOI:** 10.1039/c8ra07221c

**Published:** 2018-12-05

**Authors:** Abeer El Shahawy, Ghada Heikal

**Affiliations:** Department of Civil Engineering, Faculty of Engineering, Suez Canal University PO Box 41522 Ismailia Egypt ahmedabeer12000@yahoo.com; Environmental Engineering Department, Faculty of Engineering, Zagazig University 44519 Egypt reemhatem2006@hotmail.com +201224441973

## Abstract

Low cost adsorbents such as *P. australis* have received considerable interest owing to their low cost and easy availability. The aim of the present study was the evaluation of the removal of chemical oxygen demand (COD), biological oxygen demand (BOD), total suspended and dissolved solids (TSS and TDS) using dried *P. australis* in influent wastewater to a wastewater treatment plant. The results of the COD and BOD concentration reduction with *P. australis* at optimum operating conditions were determined for maximum reduction and adsorption isotherms. The maximum reduction of COD, BOD, TSS and TDS concentrations under the optimum operating conditions was 92.27%, 93.89%, 94.38% and 91.61%, respectively. The results demonstrate that the new dried biosorbent is able to adsorb all the aforementioned contamination. It achieved an adsorption capacity for COD of 72.5 mg g^−1^ and an adsorption capacity for BOD of 43.93 mg g^−1^. The results were well fitted by the pseudo-second order model with *R*^2^ = 0.984.

## Abstract 2

Low cost adsorbents such as *P. australis* have received considerable interest owing to their low cost, large amount of functional groups and easy availability.

## Graphical abstract

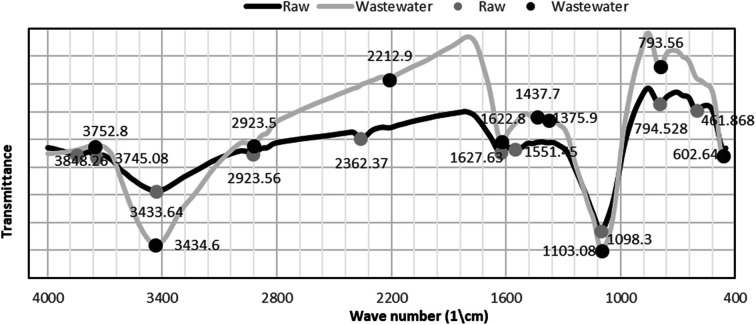

## 1. Introduction

Owing to the fast depletion of freshwater resources, the world is facing a fresh water crisis. Industrial and domestic activities have polluted the surface water as well as ground water to a greater extent.^1^ It is therefore important that a supply of good quality water should be available for various activities. However, this is becoming gradually more difficult in view of the major pollution caused by industrial, agricultural and domestic activities. These activities produce wastewater with both inorganic and organic pollutants, which result in water pollution.^2^ In rural, urbanized villages and small towns in most developing and poor countries, sewage is discharged in open drain branches. Wastewater treatment services do not exist in these areas. Wastewater collects in low lying areas or flows to open water ponds, creating a dangerous source of health threat. The conventional wastewater treatment systems are not suited for the reorganized uses for these residents. There is a critical need to develop simple, inexpensive methods and cheap strategies to treat sewage from villages, towns and cities in developing and poor countries. Chemical oxygen demand (COD), biochemical oxygen demand (BOD) and total organic carbon are measured as organic pollutants of wastewater.^3^ Wastewater treatment includes treatments such as physical, chemical and biological for removal of different pollutants from wastewater. The physical removal treatments includes screening and bar racks. The secondary treatments include biological treatments, such as activated sludge and trickling filters. Methods for organic matter removal, including various biological and non-biological methods, such as adsorption and membrane separation, can be used efficiently for removal of organic matter and heavy metals.^4^ Among these accessible procedures, adsorption is the most appealing choice for its simple application and high cost viability. Biosorption is an innovative and cost-effective biotechnology for the treatment of high-volume and low-concentration complex wastewaters and it has some advantages over some other methods for contamination removal, including high adsorption capacity and flexible operation.^5–7^

Lignocellulosic biomass derived from agricultural by-products has proven to be a promising type of raw material for producing activated carbon (AC) owing to its abundance, renewability, and rich carbon content.^8^ Stalk or stem biomass is a rich source for activated carbon production with high surface area and adsorption performance compared to that from other biomass, such as leaves or roots, owing to its high volume, low ash, high carbon, and reasonable hardness.^9^ Corn stalks,^10^ grape stalks,^11^ lotus stalks,^12^ cotton stalks,^13^ banana stalks,^14^ tobacco stems,^15^ palm stems,^16^ and giant reed stems^17,18^ have been utilized as efficient precursors for ACs with well-developed porous structures.

AC is commonly used for organic and contaminant adsorption. The majority of the past research has focused on synthetic feed models made from specific organic materials. For instance, waste materials and byproducts from agriculture and industry have been broadly used for wastewater treatment, such as biogas residues, chitosan, *Saccharomyces cerevisiae* and biomass. The vast majority of these materials are nanomaterials, which have extensive surface regions, simple change, and expansive number of dynamic destinations for overwhelming metal evacuation. However, these materials still suffer from drawbacks, such as complex preparation process, as well as difficulty in separation and regeneration.^19^ Recently, natural materials have attracted increasing attention as adsorbents for heavy metals because of their renewable and biodegradable properties, such as walnut shells, coir pith, apricot stones, almond shells, hazelnut shells, silk cotton hulls, maize cobs, coconut coir dust, peach stones, and sugarcane bagasse.^20^

*Phragmites australis* (reed) is an extensive perpetual grass found in wetlands all through calm and tropical locales of the world. *P. australis* is a warm season plant and it is well-known to have wide climatic tolerance. It can mature up to 6 m high, is seemingly perpetual and contains high levels of lignin and cellulose. The properties of *P. australis* not only give a possibly economical material for wastewater treatment, yet in addition help surface adjustment by methods for gathering extraordinary quantities of hydroxyl (OH) on its surface. Hence, *P. australis* has been utilized for quite a long time for the removal of a vast array of metals and metalloids from amphibian frameworks and wastewater.^21^*P. australis* is a very promising biosorbent because of its dense growth and its spreading root system. In addition, it has the ability to resist hostile environmental surroundings, such as alternating wet and dry conditions, elevated CO_2_ levels and high temperature. For all the previous reasons, *P. australis* is considered as one of the most effective water clearing plants for the tropics. *P. australis* has played a vital role in land reclamation.^22,23^ Although, there are different advantages of *Phragmites australis*, nearly no available studies exist in COD, BOD, TSS and TDS removal.

The best harvesting conditions can take place in the seed maturation stage and when the fine leaves become dry. To reduce insect and fungal attacks, moisture content should be eliminated as much as possible. To take benefit of the most durable part of the stem, it should be cut as close to the ground as possible. The stems with cut ends aligned are loosely tied in small bundles and combed to remove debris and fine leaves. Usually, an area is selected and all *P. australis* within that area is harvested. The cut reeds are sorted into bundles containing stems of even thickness and length. The taller and thicker stems are the most prized and of the highest quality. *P. australis* needs 1.6–2.0 hectares to cover 140 m^2^. Handling after harvest is vital. Freshly cut stems, complete with leaves, are tied into bundles and left to stand for a few days, allowing the leaves to transpire and reduce the starch content of the stem. This method, called “clump curing”, reduces attack by borer beetles, but has no effect on termites or fungi. Efficient resistance to termites, most types of fungus and fire is attained principally by chemical treatment. Dry, well-ventilated storage is essential.^24^ Reeds are one of the most frequent and dominant species in wetlands all over the world. In some cases, reed cutting can remove the N and P from the wetland, which accelerates eutrophication by pumping up nutrients from the sediments.^25^

This research mainly focused on the removal of organic pollutants from wastewater influent directly to wastewater treatment plants. The objective of the present work was to investigate the effectiveness of *Phragmites australis* as a biosorbent for the removal of COD, BOD, TSS and TDS from domestic wastewater. The focal goals of this study are to:

(i) Characterize the synthesized biosorbent by scanning electron microscopy (SEM) and Fourier transform infrared spectroscopy (FTIR).

(ii) Systematically evaluate the influences of various experimental parameters, such as biosorbent dose, contact time, pH, agitation speed and initial COD, BOD, TSS and TDS concentrations on adsorption performance.

(iii) Reveal the mechanisms of COD, BOD, TSS and TDS adsorption onto dried *Phragmites australis* biomass by determination of COD, BOD, TSS and TDS sorption kinetics, characterize COD, BOD, TSS and TDS sorption isotherms and compare sorption properties to those of other adsorbent materials. The study represents a milestone in the use of dried *Phragmites australis* biomass for future applications.

## 2. Materials and methods

### 2.1 Collection and composition of wastewater

Wastewater samples were taken from EL Keanayat wastewater treatment plant, Zagazig City, Sharkiah, Egypt after primary treatment. Wastewater can have a major adverse impact on water quality in terms of biological oxygen demand (BOD), chemical oxygen demand (COD), total suspended solids (TSS), and total dissolved solids (TDS). Table 1 displays the maximum allowable effluent discharge standards for pH, COD, BOD, TDS, and TSS (according to the Egyptian law 48/1982 for irrigation water). Additionally, the table summarizes the main characteristics of the wastewater parameters as average values, *i.e.* average measurements for readings through a year. All analyses were conducted according to the standard methods for the examination of water and wastewater. Chemical oxygen demand (COD) was measured by the closed reflux colorimetric method. Biochemical oxygen demand (BOD) was measured by the 5 days test method (see ESI S_1_†). Total suspended solids (TSS) were measured after filtration and drying at 103–105 °C. Settleable solids were measured by the gravimetric method. Total dissolved solids (TDS) were measured from the filtered liquid by the gravimetric method. The pH of aqueous solution was adjusted to 4–9 by addition of NaOH (0.1 M) or HCl (0.1 M).

**Table 1:** The characteristics of the studied wastewater parameters

Parameter	Average value	Maximum allowable^a^
pH	6.8–7	6–9
BOD (ppm)	645–655	40
COD (ppm)	1000–1100	80
TSS (ppm)	442–445	50
TDS (ppm)	828–834	2000

a Egyptian code of Environmental Regulations (1982): (4/1994).

### 2.2 Biosorbent preparation and its characterization

*Phragmites australis* (reeds) were collected from El Agoa canal in Zagazig town, situated in the Nile Delta region of Egypt. The gathered plants were analyzed, washed with 1% HCl, and after that flushed with deionized water to remove dust, residual HCL and resins attached on the plant parts. After that, the plants were placed in an oven (Shimaden) at 70 °C for 48 h to reach an oven-dry weight. Plant samples were finely crushed, grinded, and then sieved to give the desired particle size. *Phragmites australis* is a C_3_ plant, but has anatomical characteristics intermediate between C_3_ and C_4_ plants. Raw *Phragmites australis* contains, per 100 g dried biomass, about 39.5 g and 42.7 g of cellulose, 29.69 g and 27.27 g of lignin, and 23.61 g and 23.73 g of hemicellulose in the leaves and stems, respectively. The basic composition of the lignocellulosic materials is summarized in Table 2. Each experiment (refer to ESI S_2_† for the methods used for compositional analysis) was carried out in triplicate and the reported results indicate the average values of the replicated experiments. The results from the compositional analysis of raw lignocelluloses of raw *Phragmites australis* biomass (%w/w) are shown in Table 2.

**Table 2:** Compositional analysis of raw lignocelluloses from raw *Phragmites australis* biomass (%w/w)^a^

Proximate analysis (wt%)	Leaves	Stems	Lignocellulosic composition (wt%)	Leaves	Stems
Ash	4.50 ± 0.02	5.10 ± 0.026	Cellulose	39.50 ± 1.75	42.70 ± 1.83
Moisture	3.70 ± 0.15	4.20 ± 0.11	Lignin	29.69 ± 3.15	27.27 ± 2.38
Volatile	42.00 ± 0.23	36.10 ± 0.21	Hemicellulose	23.61 ± 0.52	23.73 ± 0.41
Fixed carbon	49.80 ± 0.36	54.60 ± 0.32	Extractives	7.20 ± 0.74	6.30 ± 0.89

a All values are the mean ± SD mean for three replicates.

### 2.3 Instruments

The pH was determined by using a pH meter (AD1000). All the chemicals used in the study were analytically pure and were purchased from local suppliers in Egypt. Stainless-steel sieves (Standard Sieves Dual Manufacturing Co., USA) were used to obtain the biosorbent with a definite particle size. All physicochemical analyses were performed according to the standard methods for examination of water and wastewater (APHA, 1998). The surface of the studied biosorbent, before and after the biosorption process, was analyzed and photographed using a scanning electron microscope (JEOL JSM-6510LV SEM, USA) equipped with energy dispersive X-ray spectroscopy (EDX). A Fourier transform infrared (FT-IR) spectrometer (JASCO 4100, USA) was used to detect the main functional groups responsible for organic load biosorption. The spectra were collected using an FT-IR instrument equipped with a diffuse reflectance accessory within the wavenumber range of 400–4000 cm^−1^.

### 2.4 Batch biosorption procedure

The first part of this study aimed at determining the optimum operating parameters for organic load biosorption. This included the effects of pH (4 to 9), biomass dosage level (0.5 to 3.5 g L^−1^), contact time (10 to 180 min) and agitation speed (120–300 rpm) with a plant particle size <0.15 mm on organic load removal and uptake capacity. All experiments were conducted in triplicate at a constant room temperature of 25 ± 3 °C using jar test (IMASS) apparatus for every kinetic experiment with 250 mL of domestic wastewater. The second part of this study was used to determine the effect of the initial COD, BOD, TSS and TDS concentrations by dried *P. australis* biomass. COD, BOD, TSS and TDS concentrations were varied ranging from 200 mg L^−1^ to 1100 mg L^−1^, from 100 mg L^−1^ to 655 mg L^−1^, from 89 mg L^−1^ to 445 mg L^−1^ and from 166 mg L^−1^ to 834 mg L^−1^, respectively, at the optimum adsorbent dose, contact time, pH and agitation speed identified from the first part.

### 2.5 Biosorption isotherm models

Adsorption isotherms are harmony conditions that regularly express the measure of the adsorbate on the adsorbent as a component of its concentration. The linear form of the Langmuir isotherm refers to the Langmuir equation.^26^ The logarithmic form of the Freundlich model refers to the Freundlich equation^27^ (see ESI S_3_†).

### 2.6 Kinetic studies

The adsorption rates of COD, BOD, TSS, and TDS were studied at different time intervals (10–180) min using initial concentrations of 1100, 655, 445 and 834 mg L^−1^, respectively, at the optimum adsorbent dose, pH and agitation speed. The modeling of the adsorption kinetics of COD, BOD, TSS and TDS for *P. australis* were checked by two common models, pseudo-first-order and pseudo-second-order.^28^ The details of the equations are summarized in S_3_ (see ESI S_3_†).

#### 2.6.1 The pseudo-first-order kinetic model

The pseudo-first-order model can be expressed by eqn (1-S_3_). The values of log(*q*_e_ − *q*_*t*_) were calculated from the kinetic data and plotted against time, *t*. A linear fit to the experimental data gives a straight line with a slope *k*_1_/2.303 and an intercept of log(*q*_e_) (1).1-S3
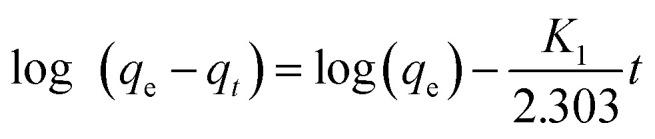
where, *q*_*t*_ is the amount of COD, BOD, TDS & TSS adsorbed at time *t* (mg g^−1^); *k*_1_ is the rate constant of pseudo-first-order in mg g^−1^ min^−1^.

#### 2.6.2 The pseudo-second-order model

The pseudo-second-order model can be expressed by eqn (2-S_3_). The values of *t*/*q*_*t*_ were calculated from the kinetic data and plotted against time, *t*. A linear fit to the experimental data should give a straight line with a slope 1/*q*_e_ and an intercept 1/(*k*_2_*q*_e_^2^) (2).2-S3
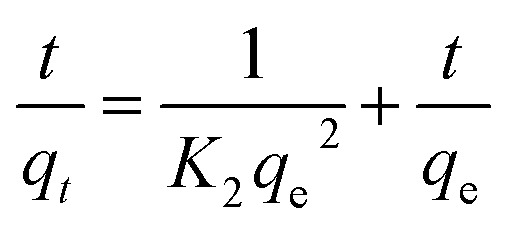
where *k*_2_ is the pseudo-second-order rate constant in g mg^−1^ min^−1^.

### 2.7 Statistical analysis

Single analysis of variance (ANOVA) and simple correlation analysis were applied to the numerical data in this study. One-way ANOVA (at a significance level of 0.05) was applied to assess the removal efficiencies of the biosorption capacity.

Interactive response surface methodology (IRSM) is a combination of statistical techniques used for designing experiments, generating models, and estimating the effects of variables. IRSM generates simulated data at combinations of independent variables specified by a designed experiment.^29^ IRSM was based on a pure-quadratic regression model, eqn (1), to fit the experimental results.

1 *Y* = *β*_0_ + *β*_1_*x*_1_ + *β*_2_*x*_2_ + *β*_3_*x*_3_ + *β*_4_*x*_4_ + *β*_5_*x*_5_ + *β*_6_*x*_1_^2^ + *β*_7_*x*_2_^2^ + *β*_8_*x*_3_^2^ + *β*_9_*x*_4_^2^ + *β*_10_*x*_5_^2^

where *Y* is the predicted response of BOD, COD, TSS and TDS removal efficiency (%); *x*_1_ is pH (4–9); *x*_2_ is initial COD, BOD, TSS and TDS concentration (200–1100, 100–655, 89–445 and 166–834 mg L^−1^, respectively); *x*_3_ is contact time (10–180 min); *x*_4_ is agitation speed (120–300 rpm); *x*_5_ is biomass dosage (0.5–3.5 g); *β*_0_ is the model intercept; *β*_1_, *β*_2_, *β*_3_, *β*_4_, and *β*_5_ are the linear coefficients of *x*_1_, *x*_2_, *x*_3_, *x*_4_ and *x*_5_, respectively; and *β*_6_, *β*_7_, *β*_8_, *β*_9_, and *β*_10_ are the quadratic coefficients of *x*_1_, *x*_2_, *x*_3_, *x*_4_ and *x*_5_, respectively.

## 3. Results and discussion

### 3.1 Characterization of raw and polluted *P. australis* dried biomass

#### 3.1.1 FTIR spectroscopy

**Fig. 1:**
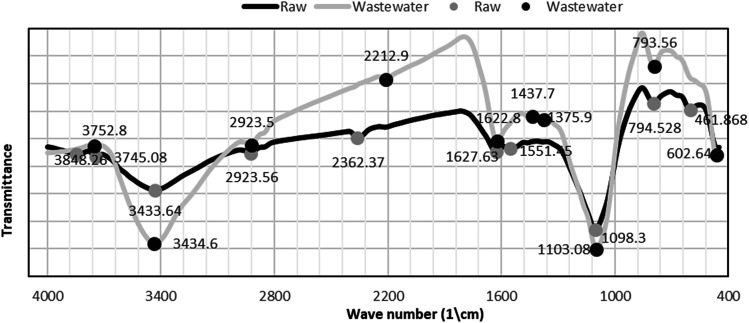
FTIR spectrum of *Phragmites australis* dried biomass before and after biosorption.

Fig. 1 shows the FTIR spectrum of *Phragmites australis* dried biomass before and after biosorption. The FTIR spectra of both the raw and adsorbent (loaded) plant biomass show that the band at 3434.6 cm^−1^ displays the distinguished broad –OH alcoholic groups of the cellulose and lignin structure.

Surface functional groups observed on *Phragmites australis* before and after COD, BOD, TSS and TDS biosorption using FTIR are illustrated in Table 3. In addition, it shows a band at 2923.5 cm^−1^ assigned to the CH stretching of methyl and methylene groups. Bands at 1627.8, 1551.45 cm^−1^ are assigned for C=C alkene and aromatics, respectively. The band at 1437.7 cm^−1^ is attributed to the CH_2_ of cellulose, hemicellulose and lignin. The band at 1375.9 cm^−1^ is for the ether group C–O. The strong band at 1098 cm^−1^ is assigned to O–H bending vibration. Bending of C=C bonds appeared at 793.5 cm^−1^. The results reveal that a large amount of chemical functional groups were preserved and generated on the surface of *P. australis*, which might enhance its adsorptive properties.

**Table 3:** Surface functional groups observed on *Phragmites australis* before and after COD, BOD, TSS and TDS biosorption using FTIR

Band position (cm^−1^)			
Before biosorption	After biosorption	Differences	Functional groups
3848.20	3752.8–3745.08	95.4	
3433.64	3434.6	−0.96	Alcohol (O–H) stretch, H-bonded
			Amine (N–H) stretch
			Amide (N–H) stretch
2923.56	2923.5	0.06	Alkane (C–H) stretch
2362.37	2212.9	149.47	Alkyne (–C=C) stretch
			Nitrile (–C≡N) stretch
1627.63	1622.8	4.83	Alkene (C=C) stretch
			Amide (N–H) bending
1551.45	1437.7–1375.9	113.75	Alkane (–C–H) bending
			Aromatic (C=C) stretch
			Nitro (N–O) stretch
			Amide (N–H) bending
1098.3	1103.08	−4.78	Alcohol (C–O) stretch
			Alkyl halide (C–F) stretch
			Amine (C–N) stretch
			Ether (C–O) stretch
			Ester (C–O) stretch
794.528	793.56	0.97	Alkene (=C–H) bending
			Alkyl halide (C–Cl) stretch
602.646	461.808	140.78	Alkyl halide (C–Cl) stretch

#### 3.1.2 Scanning electron microscopy (SEM) studies

**Fig. 2:**
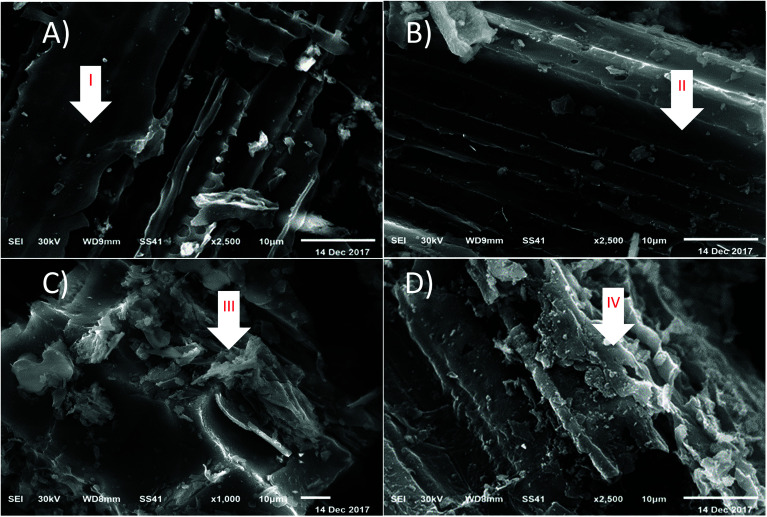
(A) and (B) SEM micrographs of raw dried *P. australis* biomass at magnification power of 2500×. (C) and (D) SEM micrographs of dried *P. australis* biomass after adsorption at magnification power of 1000× and 2500×, respectively.

The overall morphology and microscopic porous structure of the samples can be clearly seen from the SEM images, as illustrated in Fig. 2A–D. In this study, SEM photomicrographs of *P. australis* at a magnification power of 2500× showed the morphology of *P. australis* raw dried powder, as shown in Fig. 2A and B. SEM photomicrographs of the organic load and *P. australis* at a magnification power 2500× and 1000× showed the morphological changes to *P. australis* after biosorption, as shown in Fig. 2C and D. The SEM micrographs of the raw dried powder of *P. australis* show irregular particles in the range of few micrometers to few hundred micrometers. The surface of the raw dried biomass is fluffy and rough, showing a regular and compact surface structure with fibers arranged in bundles. The enhanced pictures exhibit big squares that contain channels, and the dividers of channels contain numerous bigger pores, as pointed to by arrows I and II. It can also be observed in Fig. 2A and B that the surface of the raw dried biomass is cleaner than the surface of the dried biomass after adsorption of the organic loads of COD, BOD, TSS and TDS. The surface morphology of the dried *P. australis* biomass varied and organic load molecules accumulated on the biomass surface, indicating that the organic load adsorption had occurred at the surface, as pointed to by arrows III and IV. As presented in Fig. 2C and D, the organic load particles formed aggregates of various shapes and sizes. After adsorption of COD, BOD, TSS and TDS, the pores ended up undetectable in light of the fact that the exterior biomass surface had been covered by the organic load. SEM analysis demonstrated the high affinity of *P. australis* for the immediate natural burdens, confirming the adsorption procedure.

### 3.2 Biosorbent dose

**Fig. 3:**
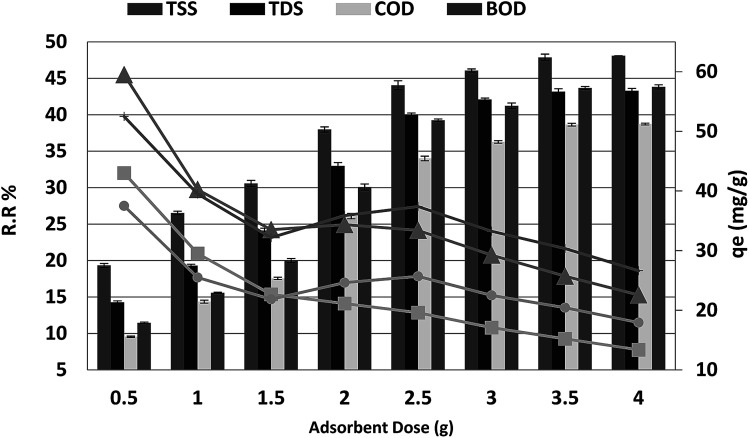
Effect of absorbent dose on the removal of efficiency and adsorption capacity for BOD, COD, TSS and TDS with a contact time of 20 min at pH 4 and agitation speed of 150 rpm.

The effect of adsorbent dosage on the percentage COD, BOD, TSS and TDS removal from 250 mL of wastewater after 20 min is shown in Fig. 3. The percentage removal of COD, BOD, TSS and TDS increased as the *P. australis* dosage increased and reached equilibrium at a dosage of 3.5 g. The increase in the adsorption of the COD, BOD, TSS and TDS by *P. australis* may be ascribed to availability of more binding sites and the high surface area. After the addition of 3.5 g, the percentage removal of COD, BOD, TSS and TDS increased rapidly from 9.55% to 38.64%, 11.45% to 43.66%, 19.33% to 47.87% and 14.47% to 43.17%, respectively, with the amount of added *P. australis* increasing from 0.5 to 3.5 g per 250 mL (*r* 0.984, *p* 0.000), (*r* 0.980, *p* 0.000), (*r* 0.981, *p* 0.000), and (0.978, 0.000) for COD, BOD, TSS and TDS, respectively. There was no significant change in the COD, BOD, TSS and TDS removal with increases in the adsorbent dose above 3.5 g, *i.e.* the percentage removal of COD, BOD, TSS and TDS was 38.73% ± 0.13, 43.82% ± 0.29, 48.09% ± 0.01, and 43.29% ± 0.34, respectively, with the added amount of *P. australis* at 4.0 g per 250 mL. This may be linked to the aggregation of binding sites and perhaps a decrease in the total adsorbent surface area of particles available to COD, BOD, TSS and TDS. Additionally, an increase in the biosorbent dose from 2 to 14.0 g L^−1^ negatively affected the *P. australis* capacity (*q*_e_) (*r* −0.768, *p* 0.044), (*r* −0.726, *p* 0.065), (*r* −0.900, *p* 0.006), and (r −0.861, *p* 0.013) for COD, BOD, TSS and TDS respectively. The *P. australis* capacity is mg of pollutant adsorbed per gram of *Phragmites australis* biomass and is calculated according to the following equation:

2 *q*_e_ = (*C*_0_ − *C*_e_) × *V*/*m*

where *V* is solution volume in liters, and *m* is the biomass weight in grams. The lowest values of 30.36, 20.43, 15.21 and 25.71 mg g^−1^ were recorded at 14.0 g L^−1^ for COD, BOD, TSS and TDS, respectively. The relationship between uptake and dosage appears to be in contrast to the trend of COD, BOD, TSS and TDS removal. This indicates a decrease in adsorption capacity per unit mass of sorbent with the increase in sorbent dosage, potentially mitigating to some extent the trend observed in increasing removal efficiency with sorbent dosage. Interference among binding sites owing to increased biosorbent dosage cannot be ruled out as this will result in low specific uptake.^30^ The optimum adsorbent dosage for COD, BOD, TSS and TDS removal from aqueous solution by *P. australis* was 14 g L^−1^.

### 3.3 Effect of contact time and adsorption kinetics

**Fig. 4:**
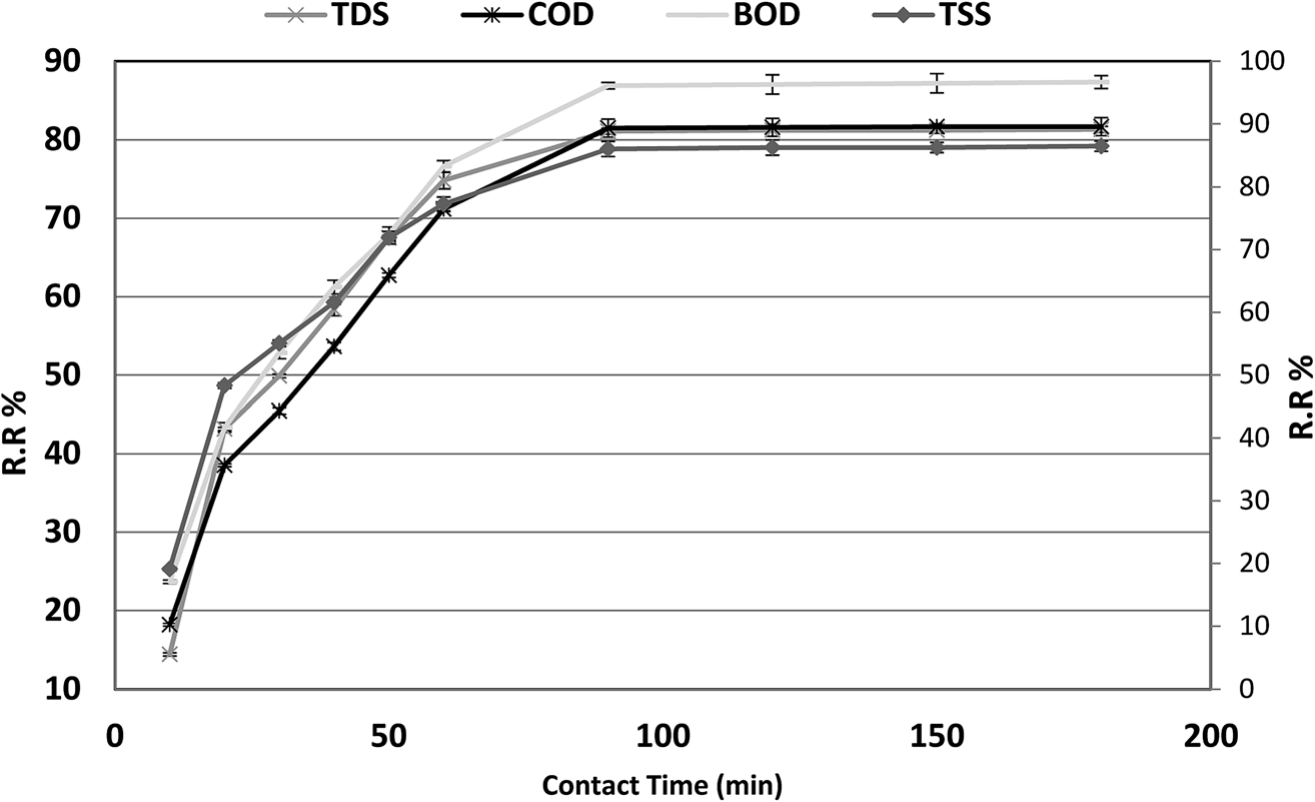
Effect of contact time on the removal of efficiency of BOD, COD, TSS and TDS at an absorbent dose of 3.5 g. pH 4, and agitation speed of 150 rpm.

**Fig. 5:**
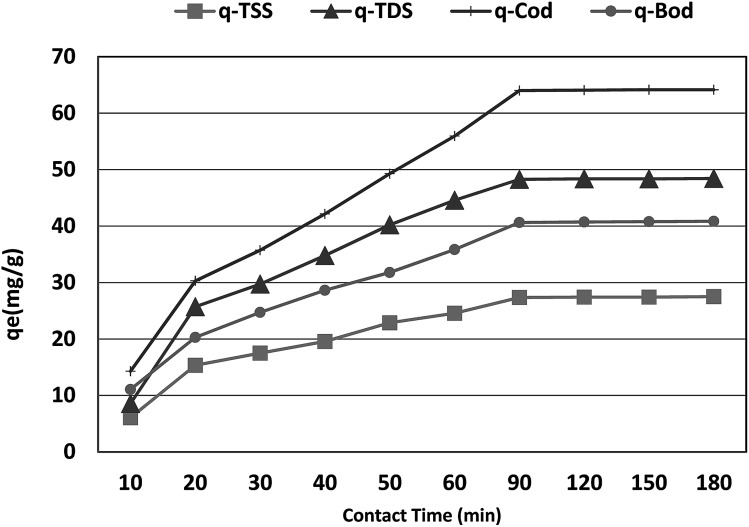
Effect of contact time on the adsorption capacity of BOD, COD, TSS and TDS at an absorbent dose of 3.5 g. pH 4, and agitation speed of 150 rpm.

The optimum contact time required for the adsorption of COD, BOD, TSS and TDS is important as it defines the time required for the adsorbates (COD, BOD, TSS and TDS) to reach an equilibrium state after contact with *P. australis*. The effect of contact time on the adsorption of COD, BOD, TSS and TDS onto *P. australis* is shown in Fig. 4 and 5.

The percent removal of COD increases with increasing contact time and biphasic kinetics are observed in each case. In the first instant, a rapid removal occurs within the first 90 min owing to a large number of available surface sites, representing the fast phase. The second stage is a slower adsorption phase process and causes smaller COD adsorption, which ended with an equilibrium point at 120 min. At this point, most of the adsorption sites were already saturated to their maximum uptake capacity. A contact time of 120 min is regarded as the optimum time for high adsorption capacity of *P. australis*. After 120 min contact time, the amount of COD, BOD, TSS and TDS removed remained constant for all the adsorbents, which might be attributed to saturation of the adsorption sites as the contact time increased. A parallel trend was found by,^31^ who stated that the biphasic mechanism basically involves external and internal diffusion processes. The maximum percentage removal of COD, BOD, TSS and TDS gradually increased from 18.18%, 23.66%, 19.10%, and 14.39% to 81.55%, 87.02%, 86.29%, and 81.18%, respectively, with an increase in contact time from 10 to 120 min, with (*r* 0.840, *p* 0.002), (*r* 0.837, *p* 0.003), (*r* 0.799, *p* 0.006) and (*r* 0.785, *p* 0.007) for COD, BOD, TSS and TDS, respectively. Similar behavior was observed for *q*_e_, which enhanced to 64.07, 40.71, 27.43, and 48.36 mg g^−1^ for COD, BOD, TSS and TDS, respectively with the contact time up to 120 min, with (*r* 0.840, *p* 0.002), (0.837, *p* 0.003), (*r* 0.799, *p* 0.006) and (*r* 0.785, *p* 0.007) for COD, BOD, TSS and TDS, respectively.

Several kinetic models, such as the pseudo-first-order, pseudo-second-order and intraparticle diffusion models, have been applied to investigate the adsorption mechanism.^28^ The equations of the two kinetic models are expressed in Table 4, where *q*_*t*_ and *q*_e_ (mg g^−1^) are the amounts of COD, BOD, TSS and TDS sorbed at time *t* and equilibrium (min), respectively. *k*_1_ and *k*_2_ (mg g^−1^ min^−1^) are the rate constants of the pseudo-first-order and pseudo-second order models, respectively. The corresponding linear parameters are presented in Table 4. Obviously, sorption of COD, BOD, TSS and TDS on *Phragmites australis* almost achieved equilibrium within the previous 120 min, indicating a high efficiency in sorbing COD, BOD, TSS and TDS.

**Table 4:** Parameters of the reaction kinetics (pseudo-first-order, pseudo-second-order) for COD, BOD, TSS and TDS biosorption using *Phragmites australis* (at room temperature of 25.00 ± 3.00 °C)

Reaction kinetics	Linear equation	Parameters	*R* ^2^
Pseudo-first-order	*Y*_COD_ = −0.0333*x* + 2.4082	*K*_l_ = 0.076 mg g^−1^ min^−1^, *q*_e_ = 255.976 mg g^−1^	0.825
	*Y*_BOD_ = −0.0305*x* + 2.1009	*K*_l_ = 0.0702 mg g^−1^ min^−1^, *q*_e_ = 126.154 mg g^−1^	0.844
	*Y*_TSS_ = −0.0288*x* + 1.8469	*K*_l_ = 0.0663 mg g^−1^ min^−1^, *q*_e_ = 70.291 mg g^−1^	0.886
	*Y*_TDS_ = − 0.0324*x* + 2.203	*K*_l_ = 0.0746 mg g^−1^ min^−1^, *q*_e_ = 159.588 mg g^−1^	0.889
Pseudo-second-order	*Y*_COD_ = 0.01261*x* + 0.4211	*K*_2_ = 0.000377 g mg^−1^ min^−1^, *q*_e_ = 79.3651 mg g^−1^	0.984
	*Y*_BOD_ = 0.0206*x* + 0.5535	*K*_2_ = 0.000767 g mg^−1^ min^−1^, *q*_e_ = 48.5437 mg g^−1^	0.992
	*Y*_TSS_ = 0.0306*x* + 0.8083	*K*_2_ = 0.001158 g mg^−1^ min^−1^, *q*_e_ = 32.6797 mg g^−1^	0.983
	*Y*_TDS_ = 0.0168*x* + 0.5288	*K*_2_ = 0.000534 g mg^−1^ min^−1^, *q*_e_ = 59.5238 mg g^−1^	0.961

The linear regression coefficients of the pseudo-second-order model were 0.984, 0.992, 0.983, and 0.961, while they were 0.825, 0.844, 0.886, and 0.889 for COD, BOD, TSS and TDS, respectively, for the pseudo-first-order model. Moreover, the equilibrium sorption amounts for COD, BOD, TSS and TDS calculated by the pseudo-second-order model were in good agreement with the experimental results as presented in Table 4. The above results indicate that sorption of COD, BOD, TSS and TDS on *Phragmites australis* could be described well by the pseudo-second-model, representing heterogeneous surface adsorption. This model clarifies the basis of the adsorption process, including the associations of valence powers or electron trade amongst the adsorbent and adsorbate.^28^ COD, BOD, TSS and TDS could be well correlated with the pseudo-second order-model representing the heterogeneous surfaces adsorption. Consequently, the sorption mechanism is ascribed to chemisorption.

### 3.4 pH effect

**Fig. 6:**
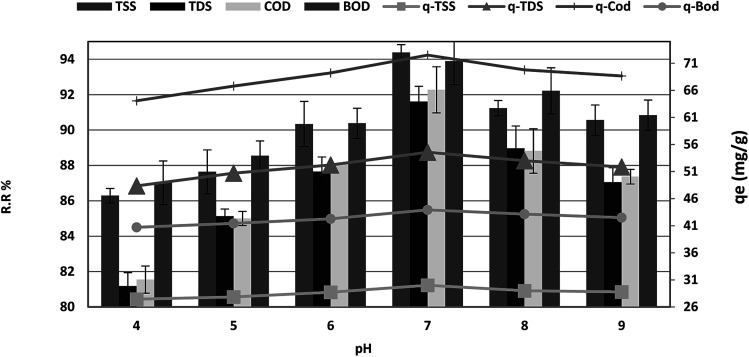
Effect of pH on the removal efficiency and the adsorption capacity of BOD, COD, TSS and TDS at an adsorbent dose of 3.5 g, contact time of 120 min, and agitation speed of 150 rpm.

The effect of pH on the adsorption of COD, BOD, TSS and TDS at an equilibrium contact time of 120 min is shown in Fig. 6. It is demonstrated that the adsorption percentage of COD, BOD, TSS and TDS onto *P. australis* increased with an increase in the pH from 4.0–7.0 and reduced in alkaline solution. This trend is explained by the competitive adsorption between aqueous H^+^ ions and COD, BOD, TSS, TDS on the surface-active sites of *P. australis* at lower pH. A positive charge causes strong electrostatic interactions between COD, BOD, TSS and TDS and *P. australis.*^32^ The amount of COD, BOD, TSS and TDS removal increased gradually from 81.55%, 87.02%, 86.29%, and 81.18% to 92.27%, 93.89%, 94.38%, and 91.61%, respectively, with an increase in pH from 4 to 7, with (*r* 0.998, *p* 0.002), (*r* 0.979, *p* 0.021), (*r* 0.976, *p* 0.024) and (*r* 0.996, *p* 0.004) for COD, BOD, TSS and TDS, respectively. Similar behavior was observed for *q*_e_, which enhanced to 72.50, 43.93, 30.00, and 54.57 mg g^−1^ with pH up to 7 with (*r* 0.998, *p* 0.002), (*r* 0.979, *p* 0.021), (*r* 0.976, *p* 0.024) and (*r* 0.996, *p* 0.004) for COD, BOD, TSS and TDS, respectively. With increasing pH, the COD, BOD, TSS and TDS sorption capacity decreased at pH values from 7.0 to 9. The COD, BOD, TSS and TDS removal ratio dropped to 87.36%, 90.84%, 90.56%, and 87.05%, with (*r* −0.973, *p* 0.147), (−0.998, *p* 0.037), (*r* −0.937, *p* 0.228) and (*r* −0.996, *p* 0.058), respectively. In the same manner, *q*_e_ dropped to 68.64, 42.5, 28.79, and 51.86 mg g^−1^ at pH 9 with (*r* −0.973, *p* 0.147), (*r* −0.998, *p* 0.037), (*r* −0.937, *p* 0.228) and (*r* −0.996, *p* 0.058) for COD, BOD, TSS and TDS, respectively. It can be seen that *P. australis* may perform well in neutral solutions but not in alkaline solutions. When the pH decreased, H^+^ ions increased in the wastewater solution and the adsorbent surface carried more negative charge, causing greater attraction between the adsorbent and the cationic form of the adsorbate. When the pH of the wastewater solution increases (pH > 7), excess OH^−^ ions for the adsorption sites produce a repulsion force towards the organic molecules' (COD, BOD, TSS and TDS) cations with negatively charged functional groups in the adsorbent surface. This could take place thus reducing the adsorption capacity.^2,33^ pH is a typical parameter in the adsorption process owing to its effect on the speciation of pollutants.^34^

### 3.5 Agitation speed effect

**Fig. 7:**
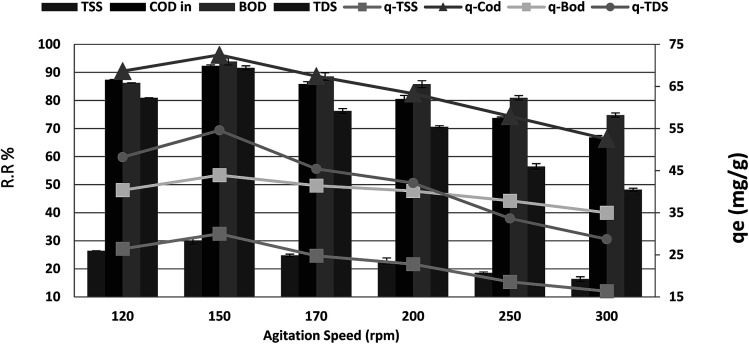
Effect of agitation speed on the removal efficiency and the adsorption capacity of BOD, COD, TSS and TDS at an adsorbent dose of 3.5 g, contact time of 120 min, and pH 7.

The maximum COD reduction was observed at 150 rpm, as shown in Fig. 7. Above 150 rpm, the loosely attached molecules might re-enter into the adsorbate, lowering the % COD reduction. Therefore, we kept the agitation speed at 150 rpm. The maximum removal efficiencies of 92.27%, 93.89%, 94.38% and 91.61% were recorded at agitation speeds from 120 to 150 rpm for COD, BOD, TSS and TDS, respectively, after which a significant decrease was noticed. When the agitation speed was increased from 150 to 300 rpm, the results were as follows: (*r* – 0.989, *p* 0.001), (*r* – 0.988, *p* 0.002), (*r* – 0.959, *p* 0.01) and (*r* – 0.970, *p* 0.006) for COD, BOD, TSS and TDS, respectively. Moreover, *q*_e_ showed a dramatic increase from 68.64, 40.36, 26.43, and 48.21 to 72.50, 43.93, 30.00, and 54.57 mg g^−1^ for COD, BOD, TSS and TDS, respectively, with an increase in agitation speed from 120 to 150 rpm. In some instances, external film diffusion can affect the biosorption process. Constant agitation can minimize this mass transfer resistance. Moreover, by increasing the agitation rate, the diffusion rate of a solute from the bulk liquid to the boundary layer liquid surrounding particles becomes higher owing to enhanced turbulence as the thickness of the liquid boundary layer decreases.^30^

However, further increase in agitation speed from 150 to 300 rpm showed a decrease in the *q*_e_ to 52.50, 35.00, 16.36, and 28.71 mg g^−1^ with the same (*r*, *p*) for the COD, BOD, TSS and TDS removal ratio, respectively. This may be attributed to the dispersal of the adsorbent particles. Because of the fact that the adsorption process is mass transfer motivated, it is thought that the liquid side mass transfer resistance controls the process. Thus, the adsorption rate increases with bulk motion.^35^ With increasing blending speed, the adsorption limit of COD, BOD, TSS and TDS diminished. This behavior was interpreted as follows: higher agitation speed lowered the biosorption capacity owing to utilization of all biosorption sites of the adsorbent. In addition, there is a higher chance that desorption could occur at high speed.^34^ This shows that the dispersion of COD, BOD, TSS and TDS particles from wastewater solution to the surface of the adsorbent and into the pores happens effortlessly and rapidly. This is because of the powder of the adsorbents. Restabilization of suspended will happen when the blending speed is quick. This phenomenon can be obviously observed for readings of blending speeds above 150 rpm.^36^*Phragmites australis* showed the most stringent changes, which proves that *Phragmites australis* is a good binder.

### 3.6 Effect of initial COD, BOD, TSS and TDS concentrations and adsorption isotherms

**Fig. 8:**
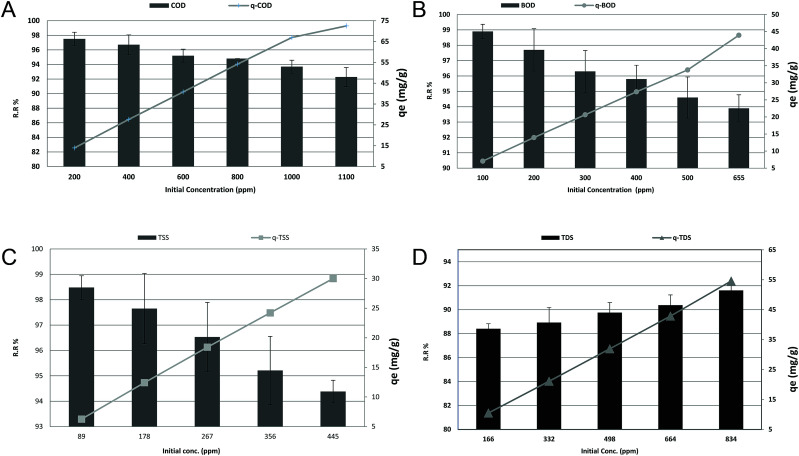
(a) Effect of initial concentration on the removal efficiency and adsorption capacity of COD at an adsorbent dose of 3.5 g, pH 7, contact time of 120 min and agitation speed of 150 rpm. (b) Effect of initial concentration on the removal efficiency and adsorption capacity of BOD at an adsorbent dose of 3.5 g, pH 7, contact time of 120 min and agitation speed of 150 rpm. (c) Effect of initial concentration on the removal efficiency and adsorption capacity of TSS at an adsorbent dose of 3.5 g, pH 7, contact time of 120 min and agitation speed of 150 rpm. (d) Effect of contact time on the removal efficiency and adsorption capacity of TDS at an adsorbent dose of 3.5 g, pH 7, contact time of 120 min and agitation speed of 150 rpm.

To evaluate the effect of COD, BOD, TSS, and TDS concentration on the adsorption efficiency removal, the adsorption process was gradually tested within the average COD, BOD, TSS and TDS concentrations in the examined wastewater, as previously displayed in Table 1, to low concentrations. The results in Fig. 8a, b and c revealed that increasing the initial COD, BOD and TSS concentrations could linearly decrease the removal ratio from 97.5%, 98.9%, and 98.48% to 92.27%, 93.89%, and 94.38%, with (*r* −0.981, *p* 0.001), (*r* −0.984, *p* 0.000) and (*r* −0.997, *p* 0.000), respectively. While, as shown in Fig. 8d, increasing the initial TDS concentration linearly increased the removal ratio from 88.41% to 91.61% with (*r* 0.989, *p* 0.001). On the other hand, the *q*_e_ increased from 13.93, 7.06, 6.26, and 10.48 to 72.5, 43.93, 30.00, and 54.57 for COD, BOD, TSS and TDS, respectively, with (*r* 1, *p* 0.00) for all. It was evident that the increase in the initial concentration of COD, BOD and TSS resulted in a decrease in the percent removal and an increase in the uptake capacity. The higher initial concentrations of COD, BOD, TSS and TDS with a fixed amount of adsorbent will result in higher equilibrium concentrations of COD, BOD, TSS and TDS in the solution, which contributes to higher amounts of COD, BOD, TSS and TDS being adsorbed by the adsorbent. At low COD, BOD and TSS loads, the number of ions competing for the existing active sites in the biomass was reduced, and therefore there was adequate surface area to lodge the COD, BOD and TSS available in the solution. In addition, the reduction of the number of available adsorption sites on the surface is one of the reasons for the reduction because the ratio of COD, BOD and TSS molecules to the adsorbent dosage rises with the elevation of the initial load. In the case of TDS, reduction of TDS, in general, has been found to increase with the increase in the initial TDS concentration. This was expected because the resistance to the diffusion of the adsorbate from the solution decreases with the increasing adsorbate load. The adsorption rate rises owing to the increasing driving force.^3^

For more information about Langmuir parameters, the reader is referred to ref. 26. As shown in Table 5, the values of *q*_max_, *K*_L_ and regression coefficient *R*^2^ are 86.9565, 33.003, 30.8642, and −116.2791 mg g^−1^, 0.03769, 0.02403, 0.18546, and −4.2744 × 10^−3^ L mg^−1^, and 0.9948, 0.9594, 0.9912, and 0.9985 for COD, BOD, TSS, TDS, respectively. A negative value for the intercept, in essence the uptake of the monolayer, is physical nonsense. It may be due to the decrease in charge density and adsorption capacity as the ionic radius increases. The *R*_L_ for COD, BOD, TSS and TDS adsorption by *Phragmites australis* was found to be 0.1171 : 0.02355 for concentrations of 200–1100 mg L^−1^ of COD, 0.03995–6.314 × 10^−3^ for concentrations of 100–655 mg L^−1^ of BOD, 0.0571–0.01197 for concentration of 89–445 mg L^−1^ of TSS and 3.44294–(−0.389886) for concentrations of 166–834 mg L^−1^ of TDS. The values of *R*_L_ for COD, BOD, TSS are in the range of 0–1, which indicates favorable adsorption, while TDS is unfavorable (*R*_L_ < 1).

**Table 5:** Parameters of the isotherm study (Langmuir and Freundlich models) for COD, BOD, oil and grease biosorption using *Phragmites australis* (at room temperature of 25.00 ± 3.00 °C)

Reaction kinetics	Linear equation	Parameters	*R* ^2^
Langmuir isotherm	*Y*_COD_ = 0.3051*x* + 0.0115	*q*_m_ = 86.9565 mg g^−1^, *K*_l_ = 0.0377 L mg^−1^	0.9948
	*R*_L COD_ = 0.1171 : 0.0235		
	*Y*_BOD_ = 0.1261*x* + 0.0303	*q*_m_ = 33.0033 mg g^−1^, *K*_l_ = 0.2403 L mg^−1^	0.9594
	*R*_L BOD_ = 0.0399 : 0.0063		
	*Y*_TSS_ = 0.1747*x* + 0.0324	*q*_m_ = 30.8642 mg g^−1^, *K*_l_ = 0.1855 L mg^−1^	0.9912
	*R*_L TSS_ = 0.0571 : 0.0120		
	*Y*_TDS_ = 2.0120*x* − 0.0086	*q*_m_ = −116.2791 mg g^−1^, *K*_l_ = −4.2744 × 10^−3^ L mg^−1^	0.9985
	*R*_L TDS_ = 3.4429 : −0.3899		
Freundlich isotherm	*Y*_COD_ = 0.5905*x* + 1.7352	1/*n* = 0.5905 L g^−1^, *K*_f_ = 5.6701 mg g^−1^	0.9920
	*Y*_BOD_ = 0.5025*x* + 1.8798	1/*n* = 0.5025 L g^−1^, *K*_f_ = 6.5522 mg g^−1^	0.9970
	*Y*_TSS_ = 0.5291*x* + 1.7114	1/*n* = 0.5291 L g^−1^, *K*_f_ = 5.5367 mg g^−1^	0.9980
	*Y*_TDS_ = 1.2364*x* + 1.3494	1/*n* = 1.2364 L g^−1^, *K*_f_ = 0.2594 mg g^−1^	0.9890

The interested reader is referred to papers by ref. 37 and 38 for more information about Freundlich equilibrium constants and their indicators. When *n* is greater than 1, the isotherm is concave downward; when *n* is less than 1, the isotherm is concave upward; and when *n* is equal to 1, the isotherm is linear. For TDS, it shows an *n* value of less than 1. Owing to the higher ionic radius of TDS, adsorption of TDS on *Phragmites australis* is very difficult and for this reason it may show less exchange capacity. Since *n* is between 1 and 10, this indicates favorable adsorption of COD, BOD and TSS onto *Phragmites australis.* As displayed in Table 5, the regression coefficients *R*^2^ are 0.992, 0.997, 0.998, and 0.989 for COD, BOD, TSS and TDS, respectively, which is regarded as a measure of the goodness of fit of the experimental data to the isotherm models. The Freundlich model is nearly coincident with the Langmuir model for the representation of the adsorption data because it has higher *R*^2^ values. Except for that, both the Freundlich model and the Langmuir model could be suitable for COD. The values of *K*_F_ and 1/*n* were 5.6701, 6.5522, 5.5367, and 0.2594 mg g^−1^ and 0.5905, 0.5025, 0.5291, and 1.2364 L g^−1^ for COD, BOD, TSS and TDS, respectively.

#### 3.6.1 Comparison with other materials

A great variety of different types of materials for COD removal have been described in previous studies. Authors such as^39–58^ listed different adsorbents employed for COD from different wastewater types, categorized as: natural materials, industrial byproducts and developed products that have been used as adsorbents for COD removal. Based on these studies, we can compare the yield of COD removal of many materials with the results obtained for the *Phragmites australis* material. Tables 6, 7 and 8 shows the efficiency reported for some materials, adsorption isotherms and kinetic studies, respectively, for removing COD from wastewater on laboratory scale. As per these examinations, materials such as chitosan flakes, avocado peel carbon and *Phragmites australis* are considered among the best in their particular classes.

**Table 6:** Comparison between the present study and previous studies for the biosorption of COD from point of view of operating parameters

Type	Material	Variables pH, temperature, COD conc., water type	Amount of COD removed (mg g^−1^)	COD removal (%)	Ref.
Composite adsorbent	Derived from rice husk ash waste (0.5 h)	pH 5, 25 °C (room temperature), 7330–9530 landfill leachate	2.2578	13.64–27.61	43
Commercial adsorbent	Activated carbon (0.5 h)	pH 5, 25 °C (room temperature), 7330–9530 landfill leachate	0.7351	24.20–36.48	
Avocado peel carbon	APC (70 min)	pH: 7, 25 °C (room temperature) initial COD and BOD: 12 000 mg L^−1^ coffee processing 24 000 plant	590.1, 297.54	COD, BOD 98.20% and 99.18%	44
	CAC (70 min)	pH: 7, 25 °C (room temperature) COD, BOD: 22 000/12 000 mg L^−1^ coffee processing plant	594.61, 298.05	COD, BOD 99.02% and 99.35%	
	Activated cow dung ash (120 minute)	pH 6.0 using 20 g L^−1^ dose in 30 °C leachate		79.0	40
	Cow dung ash (120 minute)	pH 8.0, 30 °C leachate dung ash (CA) shows 66% in 120 minutes		68	
	Charcoal	30 °C leachate		89.9	
Agricultural waste materials	Coconut shell carbon (48 h)	pH 6.0, temp. 10, 25 and 40 °C, industrial waste water		47–72	45
	Activated rice husk carbon (48 h)			45–73	
	Coconut shell carbon			50–74	
	Activated date nut carbon (180 min)	Neutral pH, ambient temperature 32 ± 1 °C, COD 100 to 800, effluent from sugar industry		73	46
	Tamarind nut carbon (180 min)			74	
	Metakaolin (180 min)			80	
	Bamboo-based activated carbon (10 h)	pH 3, 30 °C, COD 251.65, dyeing effluent from a cotton textile mill		75.21	47
Activated carbon	Rice husk (4 h)	pH 5.3, particular temperature, COD 3167, landfill leachate		70	48
	Lignite activated coke (360 min)	pH 8.483, COD = 354.6 to 67.71 mg L^−1^; temperature = 293, 303 and 313 ± 0.2 K, super heavy oil wastewater	244.7 to 43.75	63.5	49
	Activated carbon derived from peanut shells (236 min)	pH 4, 15 °C, COD 1012.6, soil eluent containing explosive contaminants	32.07	92.74	50
	1# granular activated carbon (GAC)	pH 6.28, 20 °C,	85.3		51
	2# GAC		10.4		
	RS-50B adsorption resin		136		
	Granular active carbon from lignite AC (3 h)		60.5	64.8	
	Active coke (480 min)	pH 9 to 8, 50 °C, COD 424.6, contaminated soil eluate	19.553	88.92	52
	Chitosan-coated bentonite (CCB) (20.32 h)	pH 4.0, 30 °C, COD 1348, real thin-film transistor liquid-crystal display wastewater		73.34	42
Commercial adsorbent	Walnut shell (WS) (5 minutes)	Room temperature (20 °C ± 1 °C) 1319.36 mg L^−1^ ± 4.18, synthetic produced water with different organic compounds	4.9	35.46	53
Residual biomass sources	Palm shell (PS)		5.6	28.21	
	Orange peel (OP) (5 minutes)		Discarded adsorbents	No removal	
	Banana peel (BP) (5 minutes)			No removal	
	Passion fruit peel (PP) (5 minutes)			No removal	
	Cocoa beans (CB) (5 minutes)			No removal	
	Sawdust (SD) (5 minutes)		33	23.15	
	Sodic soil	COD 8000, industrial sugar manufacture wastewater		Maximum, minimum and average COD removal: 45, 28 and 35	54
	Saline-sodic			47 and 32	
	Sugarcane bagasse fly ash (24 h)	pH from 6.5 initial to 8.82, room temperature 25 ± 1 °C, COD 5089.56, BOD1581, combined waste water sugar industry	13.94	27.4	55
				79.22	
	Marlstone	pH 5–9, room temperature (22 °C), COD 550, real wastewater from milk processing		45.7	35
Biosorbent	Water hyacinth (40 minutes)	pH 7.5–8.5, COD 2850, dairy wastewater		65.4	56
	Chitosan flakes (3 h)	pH 4.0, ambient temperature (25–30 °C), BOD, COD 1234–1968, 21, 429–31 118, biodiesel wastewater	4503, 236	90	57
				76	
Dried biomass	*Phragmites australis* (2 h)	Room temp. (25 °C) pH 7, COD 200 : 1100, BOD 100 : 655, TSS 89 : 445, TDS 166 : 834 mg L^−1^	86.9565	COD 97.5 : 92.27	This study
			33.0033	BOD 98.9 : 93.89	
			30.8642	TSS 98.48 : 94.38	
			54.57	TDS 88.41 : 91.61	

**Table 7:** Comparison between the present study and previous studies for the biosorption of COD from point of view of isotherm models

Adsorbent	Langmuir			Freundlich			Ref.
	*q*_m_ (mg g^−1^)	*K*_L_	*R* ^2^	*K*_F_	*n* ^−1^	*R* ^2^	
Chitosan-coated bentonite	47.62	0.048	0.9821	28.05	0.075	0.296	42
Walnut shell	5.638	0.0013	0.9861	0.0561	0.57	0.9972	53
Palm shell	4.924	0.0042	0.9994	0.187	0.469	0.868	
Saw dust	32.7869	0.0033	0.9896	2.2129	2.8329	0.9265	
Peanut shells	32.07	0.0022	0.823	0.13	0.813	0.954	50
*P. australis*	86.957	0.0377	0.9948	5.6701	0.5905	0.992	This study

**Table 8:** Comparison between the present study and previous studies for the biosorption of COD from point of view of kinetics models

Adsorbent	Pollutant	Model	*R* ^2^	*K*_1_ (mg g^−1^ min^−1^)	*K*_2_ (mg g^−1^ min^−1^)	*K*_id_	Ref.
Marlstone particles	COD	Pseudo-first order	0.9826	1.496	—	—	35
Marlstone particles		Pseudo-second order	0.9984	—	1.1998	—	
Peanut shells	COD	Pseudo-first order	0.806	0.0173	—	—	50
Peanut shells		Pseudo-second order	0.999	—	0.0526	—	
Supermagnetic nanoparticles	COD	Pseudo-first order	0.9661	0.00585	—	—	58
Supermagnetic nanoparticles		Pseudo-second order	0.944	—	0.0000166	—	
Palm shell	COD	Pseudo-first order	0.9919	0.3652	—	—	53
Palm shell		Pseudo-second order	0.6985	—	0.0029	—	
Walnut shell	COD	Pseudo-first order	0.9992	0.1991	—	—	
Walnut shell		Pseudo-second order	0.6616	—	0.0343	—	
Chitosan-coated bentonite	COD	Pseudo-first order	0.8661	0.17	—	—	42
		Pseudo-second order	0.998	—	0.09	—	
		Weber and Morris	0.8091	—	—	0.69	
*P. australis*	COD	Pseudo-first order	0.825	0.076	—	—	This study
		Pseudo-second order	0.984	—	0.000377	—	
		Weber and Morris	0.844	—	—	3.7032	
*P. australis*	BOD	Pseudo-first order	0.844	0.0702	—	—	
		Pseudo-second order	0.922	—	0.00767	—	
		Weber and Morris	0.841	—	—	2.7803	
*P. australis*	TSS	Pseudo-first order	0.886	0.0663	—	—	
		Pseudo-second order	0.983	—	0.001158	—	
		Weber and Morris	0.786	—	—	1.8366	
*P. australis*	TDS	Pseudo-first order	0.889	0.746	—	—	
		Pseudo-second order	0.961	—	0.000534	—	
		Weber and Morris	0.768	—	—	3.4028	

The experimental capacity of *Phragmites australis* was the fourth best after chitosan flakes, avocado peel carbon and lignite activated coke, because the amount of adsorbed COD at equilibrium decreases when the COD concentration is reduced. This resulted in a driving force induced by the reduction in the concentration gradient.

The present authors found that *Phragmites australis* dried biomass is an effective biosorbent for COD, BOD, TSS and TDS, removal exhibiting the potentiality of finding locally available materials with high pollutant maintenance limit with regards to use in wastewater treatment. *Phragmites australis* kept the first position in the capacity for COD biosorption, as indicated in Table 7 according to Langmuir, in addition to the highest removal ratio among the different adsorbents. *Phragmites australis* is very interesting and novel owing to organic pollutants in wastewater being a worldwide problem. Another important point obtained in this study is the use of clean technology, which is economical and environmentally friendly. *Phragmites australis* yields high capacity removal of all studied pollutants at the same time with the same condition with another important result of highest capacity organic pollutants removal at pH 7. Furthermore, the majority of the studies published in the literature focused on synthetic solutions with only a few studies using real wastewater.

The results revealed that the adsorption of organic contaminants on crude *Phragmites australis* was positive in different manners. For example, ease of accessibility to *Phragmites australis*, delegated amongst the most adequate feedstock for approval, simple flexibility to various environmental conditions, high biomass profitability and capacity to accelerate development. Additionally, it is a natural and cost-effective material. Preparation of dried *Phragmites australis* biomass powder is costless compared to other materials that require chemical additives. These chemical additives are ecologically unsafe. The current study demonstrated that COD removal costs by *Phragmites australis* are estimated at 0.28–0.3 $ per g COD removal. On the contrary, the COD removal costs using chitosan flakes, lignite activated coke and avocado peel carbon were estimated to be 15 and 10 $ per g COD removal, respectively, *i.e.* the cost is 30–50 times higher compared to *Phragmites australis*. Owing to the fact that few researchers have reported on TSS and TDS removal by adsorption, the efficiency comparison among different adsorbents was conducted only for COD and BOD. This was because COD and BOD are considered the main indicators of water pollution. Recently, the present authors published a study of the use of *Phragmites australis* dried biomass in oily wastewater. It achieved high capacity in retaining COD, BOD and oil and grease.^23^ It can be deduced that *Phragmites australis* achieved high efficiency in low and high initial pollutants concentrations.

### 3.7 Statistical analysis for COD, BOD, TSS and TDS

Positive linear effects of the independent variables “adsorbent dose”, and “contact time” on COD, BOD, TSS and TDS removal were observed to be significant (*p* < 0.05), as listed in Tables 9–12. Additionally, a significant negative effect (*p* < 0.05) was perceived for the quadratic term of “contact time”. However, insignificant effects (*p* > 0.05) were determined for the linear terms of “initial COD, BOD, TSS and TDS concentration”, “agitation speed”, and “pH”, as well as quadratic terms of “adsorbent dose”, “pH”, “agitation speed”, and “initial COD, BOD, TSS and TDS concentration”. The coefficient of determination between the measured data and simulated results (*r*^2^), adjusted *r*^2^, and mean squared error were (0.9883, 0.9874, 0.961, and 0.9614), (0.9835, 0.9821, 0.9441, and 0.9446) and (12.941, 13.031, 34.7331, and 35.091) for COD, BOD, TSS and TDS, respectively. The high *r*^2^ value recommended the reliability of the proposed model. For simplicity, the insignificant factors were excluded, and a new regression model was obtained, as shown in eqn (3)–(6).

3 *Y*_COD_ = 4.29 + 8.82*X*_1_ + 1.0982*X*_3_ − 0.000009*X*_2_^2^ − 0.004232*X*_3_^2^ − 0.000323*X*_4_^2^

4 *Y*_BOD_ = −2.72 + 10.94*X*_1_ + 1.1100*X*_3_ − 0.004275*X*_3_^2^ − 0.000319*X*_4_^2^ − 0.000010*X*_2_^2^

5 *Y*_TSS_ = 52.32 + 7.54*X*_1_ + 0.950*X*_3_ − 0.2678*X*_4_ − 0.003669*X*_3_^2^ − 0.000052*X*_2_^2^

**Fig. 9:**
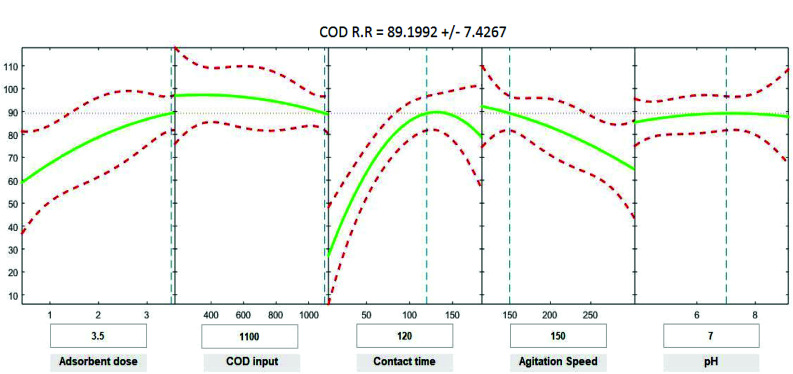
Interactive response surface methodology for estimation of COD removal efficiency using the independent variables: pH, initial COD concentration, contact time, agitation speed and adsorbent dose.

**Fig. 10:**
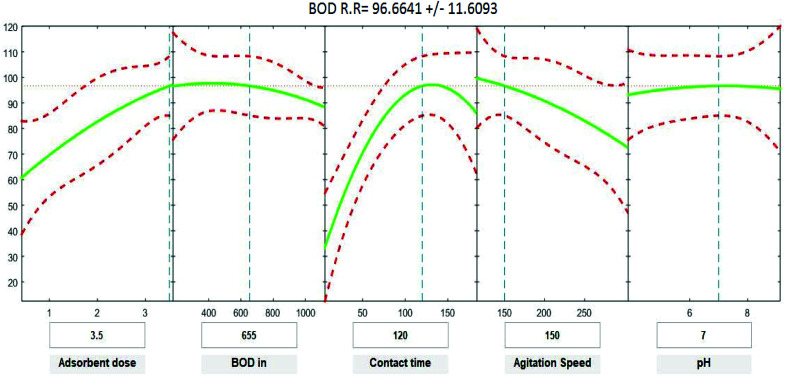
Interactive response surface methodology for estimation of BOD removal efficiency using the independent variables: pH, initial BOD concentration, contact time, agitation speed and adsorbent dose.

**Fig. 11:**
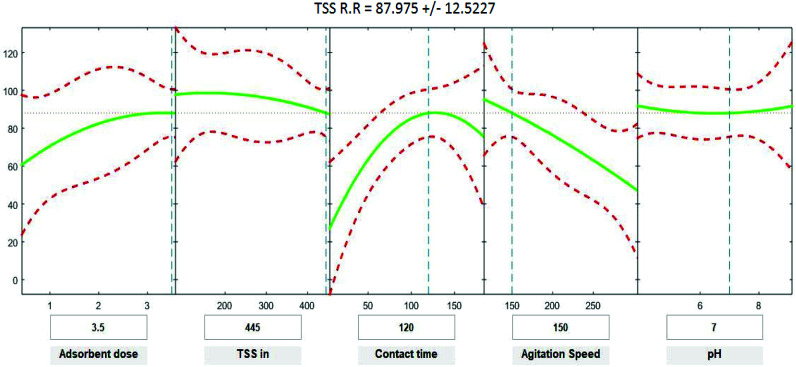
Interactive response surface methodology for estimation of TSS removal efficiency using the independent variables: pH, initial TSS concentration, contact time, agitation speed and adsorbent dose.

**Fig. 12:**
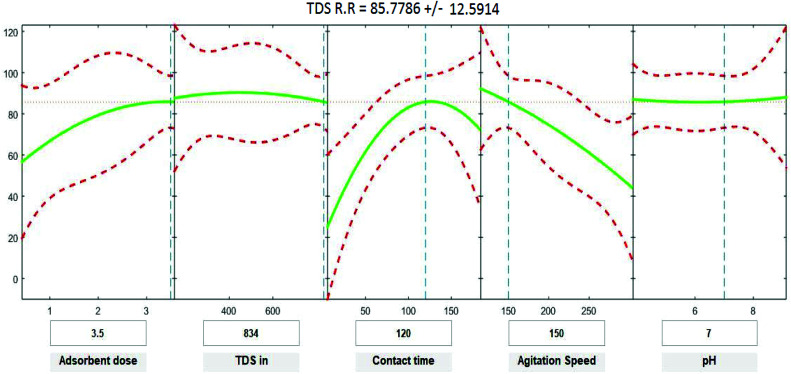
Interactive response surface methodology for estimation of TDS removal efficiency using the independent variables: pH, initial TDS concentration, contact time, agitation speed and adsorbent dose.

6 *Y*_TDS_ = 8.37 + 8.00*X*_1_ + 1.0261*X*_3_ − 0.004062*X*_3_^2^ − 0.000629*X*_4_^2^

where *Y* is the predicted response of COD, BOD, TSS and TDS removal efficiency (%); *X*_1_ is adsorbent dose (0.5–3.5); *X*_2_ is agitation speed (120–300 rpm); and *X*_3_ is contact time (10–180 min). The plot in Fig. 9–12 show a contour of IRSM for COD, BOD, TSS and TDS removal efficiency against the studied factors, pH, initial COD, BOD, TSS and TDS concentration, time, stirring speed, and biosorbent dose. The IRSM plots a 95% simultaneous confidence band for the fitted response surface as two red curves. The studied factors affecting the biosorption process are exhibited in the text boxes on the horizontal axes and are noticeable by vertical dashed blue lines in the plots. The green lines in Fig. 9–12 correspond to the pure-quadratic regression models in eqn (1) in Section 2.7. For example, at pH 7, COD, BOD, TSS and TDS concentrations of 1100, 655, 445, and 834 mg L^−1^, contact time of 120 min, agitation speed of 150 rpm, and biomass dosage of 3.5 g, the predicted COD, BOD, TSS, and TDS removal efficiency could be estimated as 89.19992%, 96.6641%, 87.975%, and 85.7786%, respectively. The COD, BOD, TSS, and TDS removal efficiency from IRSM approximately matched the actual values of 92.27%, 93.89%, 94.38%, and 91.61% under the equivalent experimental condition.

**Table 9:** *t* Statistics and *p*-values for coefficients of a pure-quadratic regression model (for COD)

	Estimate	Standard Error	*t* Ratio	Prob. > |*t*|	Effect
*β*_0_	−21.4504	24.45549	−0.87712	0.389118	Insignificant
*β*_1_	16.98549	5.864011	2.896564	0.007924	Significant
*β*_2_	0.009623	0.022559	0.426552	0.673508	Insignificant
*β*_3_	1.042557	0.077829	13.39546	1.24 × 10^−12^	Significant
*β*_4_	0.010994	0.19712	0.055774	0.955983	Insignificant
*β*_5_	5.255743	4.959888	1.05965	0.299853	Insignificant
*β*_6_	−1.82942	1.321684	−1.38416	0.179047	Insignificant
*β*_7_	−1.40 × 10^−5^	1.57 × 10^−5^	−0.89384	0.380283	Insignificant
*β*_8_	−0.00397	0.000425	−9.33486	1.85 × 10^−9^	Significant
*β*_9_	−0.00037	0.000463	−0.80724	0.427458	Insignificant
*β*_10_	−0.3677	0.40982	−0.89723	0.37851	Insignificant

**Table 10:** *t* Statistics and *p*-values for coefficients of a pure-quadratic regression model (for BOD)

	Estimate	Standard error	*t* Ratio	Prob. > |*t*|	Effect
*β*_0_	−27.6128	24.52864	−1.12574	0.271409	Insignificant
*β*_1_	17.52678	6.095145	2.875532	0.008325	Significant
*β*_2_	0.016085	0.020162	0.797794	0.432814	Insignificant
*β*_3_	1.06009	0.08001	13.24944	1.57 × 10^−12^	Significant
*β*_4_	0.012168	0.197795	0.061517	0.951457	Insignificant
*β*_5_	4.532874	4.929535	0.919534	0.366967	Insignificant
*β*_6_	−1.49156	1.421889	−1.049	0.304628	Insignificant
*β*_7_	−1.91 × 10^−5^	1.36 × 10^−5^	−1.40589	0.172573	Insignificant
*β*_8_	−0.00404	0.000432	−9.33989	1.83 × 10^−9^	Significant
*β*_9_	−0.00037	0.000465	−0.79977	0.431687	Insignificant
*β*_10_	−0.31415	0.407606	−0.77072	0.448394	Insignificant

**Table 11:** *t* Statistics and *p*-values for coefficients of a pure-quadratic regression model (for TSS)

	Estimate	Standard error	*t* Ratio	Prob. > |*t*|	Effect
*β*_0_	42.63803	40.51244	1.052468	0.303517	Insignificant
*β*_1_	21.73281	9.60698	2.26219	0.033442	Significant
*β*_2_	0.041681	0.099458	0.419078	0.679047	Insignificant
*β*_3_	1.04609	0.127516	8.203627	2.78 × 10^−8^	Significant
*β*_4_	−0.13051	0.322946	−0.40413	0.689852	Insignificant
*β*_5_	−7.29601	8.185479	−0.89134	0.381979	Insignificant
*β*_6_	−3.27631	2.165309	−1.51309	0.143877	Insignificant
*β*_7_	−0.00013	0.000169	−0.77049	0.448854	Insignificant
*β*_8_	−0.00409	0.000696	−5.87471	5.48 × 10^−6^	Significant
*β*_9_	−0.0003	0.000759	−0.39118	0.699264	Insignificant
*β*_10_	0.558893	0.675577	0.827282	0.416578	Insignificant

**Table 12:** *t* Statistics and *p*-values for coefficients of a pure-quadratic regression model (for TDS)

	Estimate	Standard error	*t* Ratio	Prob. > |*t*|	Effect
*β*_0_	9.378485	40.71209	0.230361	0.819848	Insignificant
*β*_1_	22.33025	9.656334	2.312497	0.030047	Significant
*β*_2_	0.027089	0.053133	0.509829	0.615029	Insignificant
*β*_3_	1.057796	0.128171	8.253026	2.50 × 10^−8^	Significant
*β*_4_	−0.062	0.324605	−0.191	0.850202	Insignificant
*β*_5_	−3.35297	8.22849	−0.40748	0.687421	Insignificant
*β*_6_	−3.27465	2.176433	−1.5046	0.146038	Insignificant
*β*_7_	−3.02 × 10^−5^	4.83 × 10^−5^	−0.62578	0.53762	Insignificant
*β*_8_	−0.00419	0.0007	−5.98465	4.21 × 10^−6^	Significant
*β*_9_	−0.00046	0.000763	−0.60708	0.549747	Insignificant
*β*_10_	0.272288	0.679115	0.400945	0.692161	Insignificant

## 4. Conclusion

The present study concluded that FTIR results reveal that a large amount of chemical functional groups were preserved and generated on the surface of *P. australis*, which might enhance its adsorptive properties owing to the functional groups –OH, CH_2_, C–O and C=C. The SEM analysis showed confirmation of the adsorption process owing to the surface texture of the examined plant. This study demonstrated that *P. australis* can reduce the organic load (COD, BOD, TSS and TDS) in domestic wastewater and achieved higher removal ratios compared to other agro-wastes. It was also found that a large amount of chemical functional groups were conserved on the surface of *P. australis*, with great biosorption capacity of COD, BOD, TSS and TDS at pH 7. The batch adsorption study showed that the removal of COD was well-matched with the Langmuir isotherm model. This demonstrates that the adsorption occurred by monolayer adsorption because of particular holding amongst the adsorbate and the surface of the adsorbent. On the other hand, the removal of COD, BOD, TSS and TDS was well-fitted with the Freundlich isotherm model, which indicates multilayer adsorption taking place on the innumerous heterogeneous *P. australis* surface adsorption sites. This was also supported by the results of the kinetic study for COD, BOD, TSS and TDS removal, which were obtained following the pseudo second-order model. This model, used to clarify the probability of overall adsorption properties, was suited to the chemical adsorption mechanism. Finally, IRSM was employed for modeling and optimizing COD, BOD, TSS and TDS biosorption on *P. australis*. The nature of water after treatment was observed to be appropriate for water system utilization and for coordinated release into streams.

## Conflicts of interest

The authors have no conflict of interest.

## Supplementary Material

RA-008-C8RA07221C-s001
