# Supplementary material for: Regression, kinetics and isotherm models for biosorption of organic pollutants, suspended and dissolved solids by environmentally friendly and economical dried Phragmites australis
Source: RSC Adv. 2018 Dec 5;8(71):40511–28. doi: 10.1039/c8ra07221c (PMC9091435; doi:10.1039/c8ra07221c)
Supplement: RA-008-C8RA07221C-s001 [file RA-008-C8RA07221C-s001.pdf]

Electronic Supplementary Material (ESI) for RSC Advances.
This journal is © The Royal Society of Chemistry 2018

1-Supplementary (S_1)

1.Biological Oxygen Demand (BOD)

Test Procedure

The **BOD** test takes **5 days to complete** and is performed using a dissolved oxygen test kit. The **BOD** level is determined by comparing the **DO** level of a water sample taken immediately with the **DO** level of a water sample that has been incubated in a dark location for **5 days**. The difference between the two **DO** levels represents the amount of oxygen required for the decomposition of any organic material in the sample and is a good approximation of the **BOD** level.

1-Take **200ml**of water sample and **70ml** of aerated water in **BOD** bottle.

(The sample needed to be diluted with aerated water to give the amount of oxygen needed for decomposition of organic materials in the water by microorganisms).

2-Take **270 ml** of aerated water in **BOD** bottle as blank sample (two blank bottles).

3-Record the **DO** level (ppm) immediately using the dissolved oxygen prob.

4-Place the water sample and the blank samples in an incubator in complete darkness at **20C** for **5days**.

5- After **5 days**, take another dissolved oxygen reading (ppm) using the dissolved oxygen prob.

6- **BOD** value is calculated by the following equation:

$$\textbf{BOD} = \frac{Difference\ in\ DO - Error * 270}{volume\ of\ sample}$$

- **Difference in DO:**is the difference in the **DO** value between **before** and **after** incubation.
- **Error:** is the difference in the **DO** value between the two **blank** samples.
- **270**: is the total volume of water in the BOD bottle.

2. chemical oxygen demand (COD) mg/l

Closed Reflux, Colorimetric Method

COD test is based on the principal that most organic compounds are oxidized to CO_2 and H_2O by storing oxidizing agents under acid conditions. COD was measured using closed flux methods. COD concentrations were determined colorimetrically. A total of 2.5 ml

samples were added to COD reaction vials, containing 3.5 ml of silver sulphate/ sulphuric acid and 1.5 ml digestion solution and digested at 150°C for 2 hours. A blank sample containing distilled water was also prepared. After digestion, vials were allowed to cool, inverted twice and allowed to stand before measuring absorbance at 600 nm. The spectrophotometer was zeroed using the blank vial.

1 Apparatus

Digestion vessels 15 x 180 mm with screw caps.

Oven to operate at 150 ± 2°C.

Spectrophotometer for use at 600 nm

2 Reagents

a) Digestion solution: Add to about 500 mL H_2O 10.216 g $K_2Cr_2O_7$, previously dried at 103°C for 2 hours, 167 mL conc. H_2SO_4 and 33.3 g $HgSO_4$. Dissolve (this will take many hours, mixing during this time is not required, however mix after dissolving), cool to room temperature and dilute to 1000 mL

b) H_2SO_4/Ag_2SO_4: Add 10 g Ag_2SO_4 to 1 L conc. H_2SO_4, let stand overnight to dissolve. Mix carefully after dissolving

c) Stock KHP: 850 mg potassium hydrogen phthalate, dried at 120°C for 24 hours, is dissolved in 1000 mL H_2O.

The COD of this stock solution is 1000 mg O_2/L.

3 Preparation of standards

Prepare standard solutions containing a known COD concentration by diluting a known volume, A, of the stock KHP to 100 mL.

mL A/100 mL	COD mg/L
0	0
2	20
5	50
7	70
10	100
20	200
30	300
40	400
50	500
60	600
70	700
80	800
90	900

4 Procedure

Wash the digestion tubes and caps with 4 M H2SO4 before first use to prevent contamination.

1. Transfer 2.5 mL of standard or sample to the digestion tube and add 1.5 mL digestion solution
2. Carefully run 3.5 mL H_2SO_4/Ag_2SO_4 down inside of tube so an acid layer is formed under the sample-digestion solution layer. Tightly cap tube and swirl several times to mix completely, do not invert the tubes!
3. Place the tubes in a preheated oven of 150°C during 2 hours
4. Allow them to cool, mix the content and let particles settle
5. The next day: Transfer the content gently and without mixing to a 1 cm cell and measure the absorbance at 600 nm against water
6. Plot the absorbance against the known COD in order to get a calibration line.
7. Read absorbance of samples and compare to calibration line, determine the mathematical equation of this line.

Remarks:

1. Use an adjustable Eppendorf pipette
2. Run the standard and samples in duplicate
3. For each set of samples also analyses simultaneously one or two standards with a COD value comparable to the samples.

References

1. Rice, E.W., Bridgewater, L., American Public Health, A., American Water Works, A. and Water Environment, F. (2012) Standard methods for the examination of water and wastewater, American Public Health Association, Washington, D.C.

Supplementary (S_2)

1. Characterization of the dried biomass

The following characteristic properties of the dried were studied

1.1Proximate Analysis-

ASTM defines proximate analysis as the determination by prescribed methods of moisture, volatile matter, ash & fixed carbon. The proximate analysis of the givenactivated carbon sample will be followed by the procedure given below.

a) Moisture Content-

A small amount of the sample of the dried biomass was put in a petri-dish or crucible, covered with a lid &weighed using a weighing balance. The crucible was placed in the hot air oven at 105° C withits lid removed & dried for 1.30 hrs. The crucible was taken out, immediately covered withthe lid, cooled in a desiccator& weighed.

$$M=100(B-F)/(B-G) \quad \text{Eq. 1-}S_2$$

Where:

Wt of empty petri-dish =G

Wt of empty petri-dish + Activated carbon sample (before heating)= B

Wt of empty petri-dish + moisture free sample (after heating)= F

b) Ash content

The crucible was ignited in the muffle furnace at 750+ 25°C for 1.5 hours. Thecrucible was placed in the desiccator, cooled to room temperature & weighed. A knownamount of the sample of the dried biomass was dried in the hot air oven at 150°C for 3 hours was put inthe crucible & the crucible was placed back in the muffle furnace at 750+25°C for 1.5hours. The crucible was taken out of the furnace, placed in the desiccator, cooled to roomtemperature & weighed.

A=100(F-G)/(B-G) **Eq. 2-S_2**

Where:

Wt of empty silica crucible = G

Wt of empty crucible + activated carbon sample (before heating) = B

Wt of empty crucible + ash (after heating) = F =

c) Volatile matter content

A known amount of sample of the dried biomass was put in the crucible. The crucible was placed in amuffle furnace at 920+10°C, covered with lid, & placed for exactly 7 minutes. The cruciblewas taken out, allowed to cool & weighed.

VM=100(100(B-F)-M(B-G))/((B-G) (100-M) **Eq. 3-S_2**

Where:

Wt of empty crucible with lid = G

Wt of empty crucible + lid + sample(before heating) = B

Wt of empty crucible + lid + sample (after heating) = F

M=Moisture content in %

d) Fixed carbon

% of fixed carbon=100-(M+A+V M) **Eq. 4-S_2**

2. Compositional analysis of the raw lingo-cellulosic materials: the biomass materials were subjected to compositional analysis using the gravimetric method. Materials used in this study were all unscreened.

a) Extractives: 2.5 g of dried raw biomass was loaded into the cellulose thimble. With the Soxhlet extractor set up, 150 mL of acetone was used as solvent for extraction. Residence times for the boiling and rising stages was carefully adjusted to 70°C and 25 min respectively on the heating mantle for a 4 h run period. After extraction, the sample was air dried at room temperature for few minutes. Constant weight of the extracted material achieved in a convection oven at 105°C. The %(w/w) of the extractives content was evaluated as the difference was in weight between the raw extractive-laden biomass andextractive-free biomass.[1-3]

b) Hemicellulose: 1 g of extracted dried biomass was transferred into a 250 mL Erlenmeyer flask. 150 mL of 500 mol/m^{3}NaOH was added. The mixture was boiled for 3.5 h with distilled water. It was filtered after cooling through vacuum filtration and washed until neutral pH. The residue was dried to a constant weight at 105°C in a convection oven. The difference between the sample weight before and after this treatment is the hemicellulose content (%w/w) of dry biomass.[1-4]

c)Lignin: 0.3 g of dried extracted raw biomass was weighed in glass test tubes and 3 mL of 72% H_2SO_4 wasadded. The sample was kept at room temperature for 2 h with carefully shaking at 30 min intervals to allow for complete hydrolysis. After the initial hydrolysis, 84 mL of distilled water was added. The second step of hydrolysis was made to occur in an autoclave for 1 hat 121 °C. The slurry was then cooled at room temperature. Hydrolyzates were filtered through vaccum using a filtering crucible. The acid insoluble lignin was determined by drying the residues at 105°C and accounting for ash by incinerating the hydrolyzed samples at 575°C in a muffle furnace. The acid soluble lignin fraction was determined by measuring the absorbance of the acid hydrolyzed samples at 320 nm. The lignin content was calculated as the summation of acid insoluble lignin and acid soluble lignin.[5]

d) Cellulose: The cellulose content (%w/w) was calculated by difference, assuming that extractives,hemicellulose, lignin, ash, and cellulose are the only components of the entirebiomass.[1-3]

References

1 **C. D. Blasi, G. Signorelli, C. Di Russo, G. Rea,** IndustrialEngineering Chemistry Research, 38(6), **1999, 2216-2224.**

2 **S. Li, S. Xu, S. Liu, C. Yang and Q Lu,** Fuel ProcessTechnology, 85(8–10), **2004, 1201-1211.**

3 **L. Lin, R. Yan, Y. Liu and W. Jiang,** Bioresource Technology, 101(21), **2010, 8217-8223.**

4 **A. O. Ayeni, F. K. Hymore, S. N. Mudliar, S. C. Deskmukh, D. B. Satpute, J. A. Omoleye and R. A. Pandey,** Fuel, 106, **2013, 187-194.**

5 **A. Sluiter, B. Hames, R. Ruiz, C. Scarlata, J. Sluiter and D. Templeton et al., CO: National Renewable Energy Laboratory; April 2008.NREL Report No.: TP-510-42618. Contract No.:DE-AC36-99-G010337. Sponsored by the U.S. Department of Energy.**

2- Supplementary (S_3)

Biosorption kinetics

The pseudo-first-order kinetic model

Pseudo-first-order model can be expressed by **Eq. (1-S_3)**. The values of log ($q_e - q_t$) were calculated from the kinetic data and plotted against time t. A linear fit to the experimental data gives a straight line with a slope $k_1/2.303$ and an intercept log(q_e).[1]

$$\text{Log}(q_e - q_t) = \log(q_e) - \frac{K_1}{2.303}t \qquad \textbf{Eq.1-S}_3$$

Where, q_t is the amount of COD, BOD and Oil& Grease adsorbed at time t (mg g^{-1}); k_1 is the rate constant of pseudo-first-order in mg g^{-1} min^{-1}.

The pseudo-second-order model

Pseudo-second-order modelcan be expressed by **Eq. (2-S_3)**. The values of t/q_t were calculated from the kinetic data and plotted against time t. A linear fit to the experimental data should give a straight line with a slope $1/q_e$ and an intercept 1/ ($k_2.q^2{}_e$).[2]

$$\frac{t}{q_t} = \frac{1}{K_2.q_e^2} + \frac{t}{q_e} \qquad \textbf{Eq.2-S}_3$$

Where, k_2 is the rate constant of pseudo-second-order in g mg^{-1} min^{-1}.

Isotherm studies

Langmuir isotherm

As formulated in **Eq. 4-S_2**, the linear plot of C_e/q_e versus C_e gives a slope $1/q_m$ and an intercept $1/(q_m.K_L)$.[3]

$$\frac{C_e}{q_e} = \frac{1}{q_m.K_L} + \frac{C_e}{q_m} \qquad \textbf{Eq. 3-S}_3$$

Where, q_m is the maximum sorbate uptake under the given condition, also known as the monolayer biosorption capacity (mg g^{-1}); K_L is the Langmuir biosorption coefficient related with the free energy of sorption (L mg^{-1}).

In addition, the dimensionless separation factor constant (R_L), can be estimated from the Langmuir isotherm by Eq. **(4-S$_3$)**.

$$R_L = \frac{1}{1 + K_l C_0} \qquad \textbf{Eq. 4-S}_3$$

Where C_0 is the initial concentration of BOD, COD and oil & grease. Values of $R_L > 1$ and $0 < R_L < 1$ show unfavorable and favorable adsorptions, respectively, while values of $R_L = 1$ and $R_L = 0$ show a linear adsorption and irreversible adsorption, respectively.

Freundlich isotherm

Freundlich model can be applied to multilayer adsorption, taking place on the heterogeneous adsorbent surface with different adsorption sites.[4] As formulated in **Eq. (6-S$_2$)**, a linear plot of ln (q_e) versus ln (C_e) gives a slope 1/n and an intercept ln(K_F).[5]

$$\ln (q_e) = \ln (k_f) + \frac{1}{n} \ln(C_e) \qquad \textbf{Eq. 5-S}_3$$

Where,

K_F is a constant relating the biosorption capacity (the ability of biosorbent to adsorb); 1/n is the heterogeneity factor of the adsorbent, relating the biosorption intensity: the closer 1/n values to zero, the more heterogeneous the adsorption surface.

References

1 S. Lagergren, Handlingar, 1898, 24 (4), 139.

2 Y. S. Ho, G. Mckay, Pseudo-second order model for sorption processes, Process Biochemistry 34, (1990) 451-465.

3 I. Langmuir, J. Am. Chem. Soc., (September 1918), 40 (9), 1361–1403. DOI: 10.1021/ja02242a004.

4 S. Cataldo, G. Cavallaro, A. Gianguzza, G, Lazzara, A. Pettignano, D. Piazzese, I. Villaescusa, Journal of Environmental Chemical Engineering, 2013 1(4), 1252-1260.

5 Freundlich, H., 1906. Adsorptionin solution [j]. Phys Chem Soc 40, 1361-1368.
